# Pre-operative apparent diffusion coefficient values and tumour region volumes as prognostic biomarkers in glioblastoma: correlation and progression-free survival analyses

**DOI:** 10.1186/s13244-019-0724-8

**Published:** 2019-03-18

**Authors:** Coral Durand-Muñoz, Eduardo Flores-Alvarez, Sergio Moreno-Jimenez, Ernesto Roldan-Valadez

**Affiliations:** 1 Department of Internal Medicine, Medica Sur Clinic and Foundation, Mexico City, Mexico; 2 Department of Neurosurgery, Secretariat of Health, General Hospital of Mexico, Mexico City, Mexico; 3 Radioneurosurgery Unit, The National Institute of Neurology and Neurosurgery, Mexico City, Mexico; 4 Directorate of Research, Secretariat of Health, General Hospital of Mexico, Mexico City, Mexico; 5 Department of Radiology, I.M. Sechenov First Moscow State Medical University (Sechenov University), Trubetskaya str., 8, b. 2, Moscow, Russia 119992

**Keywords:** Brain neoplasms, Diffusion magnetic resonance imaging, Glioblastoma, Neuroimaging, Regression analysis, Progression-free survival

## Abstract

**Objectives:**

Glioblastoma (GB) contains diverse histologic regions. Apparent diffusion coefficient (ADC) values are surrogates for the degree of number of cells within the tumour regions. Because an assessment of ADC values and volumes within tumour sub-compartments of GB is missing in the literature, we aimed to evaluate these associations.

**Methods:**

A retrospective cohort of 48 patients with GB underwent segmentation to calculate tumour region volumes (in cubic centimetre) and ADC values in tumour regions: normal tissue, enhancing tumour, proximal oedema, distal oedema, and necrosis. Correlation, Kaplan-Meier, and Cox hazard regression analyses were performed.

**Results:**

We found a statistically significant difference among ADC values for tumour regions: *F* (4, 220) = 166.71 and *p* ≤ .001 and tumour region volumes (necrosis, enhancing tumour, peritumoural oedema): *F* (2, 141) = 136.3 and *p* ≤ .001. Post hoc comparisons indicated that the only significantly different mean score was the peritumoural volume in oedema region (*p* < .001). We observed a positive significant correlation between ADC of distal oedema and peritumoural volume, *r* = .418, df = 34, and *p* = .011. Cox proportional hazards regression analysis considering only tumour region volumes provided an almost significant model: − 2 log-likelihood = 146.066, *χ*^2^ (4) = 9.303, and *p* = .054 with a trend towards significance of the hazard function: *p* = .067 and HR = 1.077 for the non-enhancing tumour volume.

**Conclusions:**

ADC values together with volumes of oedema region might have a role as predictors of progression-free survival (PFS) in patients with GB; we recommend a routine MRI assessment with the calculation of these biomarkers in GB.

## Key points

- In GBM, there is a statistically significant difference among ADC values for tumour regions.
- In GBM, there is a statistically significant difference among tumour region volumes (necrosis, enhancing tumour, and peritumoural oedema).
- ADC of distal oedema and peritumoural volume depict a positive significant correlation.
- The non-enhancing tumour volume depicts a trend towards significance (*p* = .067, HR = 1.077) for progression-free survival.
- A routine MRI assessment with the calculation of ADC and volume biomarkers in GBM might be performed on a day-to-day basis.

## Introduction

Glioblastoma (GB) is the most frequent primary brain tumour containing various histologic regions [1]. Therefore, a sampling error in a biopsy implies that the degree of severity assessed by the pathologist does not reveal the actual degree of malignancy present elsewhere in a tumour [2]. Pre-surgical prognostic factors often include performance status, age, the extent of resection, and O^6^-methylguanine-DNA methyltransferase (MGMT) methylation status [3]. Malignant GB cells might arise from the transformed subventricular zone (SVZ), the astrocyte-like neural stem cells, while others initiate progression to malignancy of the non-SVZ progenitor cells or mature glial cells that have undergone dedifferentiation [4].

Magnetic resonance (MR) protocols for GB in a day-to-day practice usually include four morphologic sequences: T1 pre-gadolinium, T1post-gadolinium, T2, and FLAIR [5]. Surgery usually only reduces the surgical and radiologically visible portions of a tumour. Then, some surgical margins may not be “clean” (due to the presence of microscopic residual lesions) which leads to further neoplastic growth in adjacent brain tissue (gross recurrence) [6]. Advanced MR imaging techniques using diffusion-weighted imaging (DWI) allows the calculation of DWI-derived apparent diffusion coefficient (ADC) to characterise GB [7]. ADC values are currently accepted as surrogates for the level of cellularity within a tumour [3]; a low ADC preoperatively has shown a negative correlation with the Ki-67 labelling index and can predict the progression in malignant astrocytomas [8]. Also, preoperative ADC has shown an adverse prognostic factor independent of performance status, age, and the resection volume [9].

Recent advances in brain tumour image analysis allow an automatic segmentation of tumour sub-compartments from magnetic resonance imaging (MRI) by using the conventional MRI sequences T1-w, T1-w post contrast administration, T2-w, and FLAIR [10]. It is possible to obtain tumour region volumes in cubic centimetres of the necrotic tissue, active enhancing tumour tissue, non-enhancing tumour tissue, and oedema; the definition of these tumour sub-compartments follows the VASARI guidelines of the National Cancer Institute [11]. Some authors have classified GB based on its involvement of the SVZ and cortex; this classification considers the histogenetic and clinical heterogeneity of GB [12]. Although previous information exists, correlation values between ADC and tumour region volumes in GB are still missing in the literature.

In this study, we aimed to reveal the associations of ADC values and volumes within tumour sub-compartments of GB; furthermore, we evaluated the ability of ADC pre-treatment values and the MRI-based classification of GBs involvement of the SVZ as biomarkers of the progression-free survival.

## Methods

### Patient selection

The Institutional Review Board of The National Institute of Neurology and Neurosurgery and the General Hospital of Mexico approved the study, and patients received and gave informed consent before they underwent MRI studies. A retrospective cohort of 48 patients with glioblastoma multiforme underwent follow-up; patients were included from January 2011 to December 2013. The group consisted of 36 males: 44.6 ± 17.8 (mean and standard deviation [SD]), range 18–70 years and 12 females: 50.5 ± 12.6, range 18–66 years. Exclusion criteria considered surgical resection, radiotherapy, or chemotherapy previous to the inclusion in the study, absent histopathologic diagnoses, missing imaging data, and presence of artefacts. After resection, they received chemotherapy including temozolomide (Temodal, Schering-Plough, NJ, USA) and radiation therapy (60 Gy), the Stupp protocol [13]. All patients underwent biopsy or surgical resection of a tumour with histopathologic diagnosis based on the WHO criteria. MRI image interpreters were blinded to the patient’s history. MRI examinations with other structural abnormalities were removed.

### Brain image and data acquisition

**Fig. 1:**
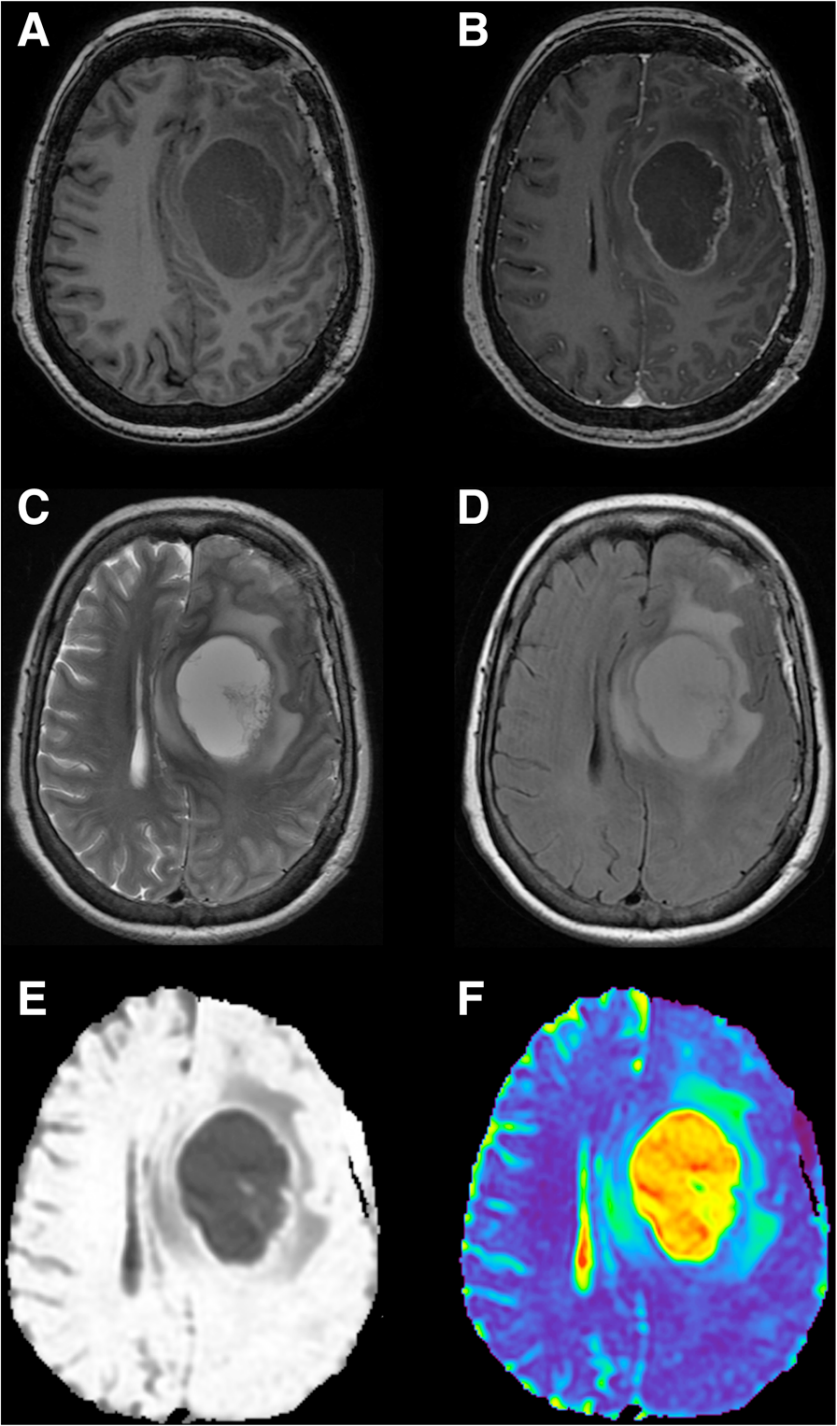
Conventional MR sequences in the axial plane for the assessment of GB. **a** T_1_-weighted imaging. **b** Post-gadolinium T_1_-weighted imaging. **c** T_2_- weighted imaging. **d** Fluid-attenuated inversion recovery (FLAIR). **e** Diffusion-weighted imaging (DWI). **f** DWI-derived mapping with the calculation of apparent diffusion coefficient values

MR images were acquired by using a 3T scanner (Signa HDxt, GE Healthcare; Magnetic Resonance Unit; Waukesha, Wisconsin, USA) with a high-resolution eight-channel head coil (Invivo, Gainesville, FL, USA). MR sequences included conventional axial T_2_-w, axial fluid-attenuated inversion recovery (FLAIR), axial spoiled gradient echo (SPGR), diffusion-weighted imaging (DWI), and axial T_1_-w, using 0.1 mmol/kg of body weight of gadopentetate dimeglumine (Magnevist; Schering, Berlin, Germany). DWI was performed using a single-shot SE-EPI sequence with *b* values 1000 s/mm^2^ and 0 s/mm^2^. Figure 1 shows the appearance of conventional MRI sequences in a patient with GB.

### Segmentation of tumour regions and volume calculations

We used BraTumIA software (v 1.2.1© 2018 Institute for Surgical Technology and Biomechanics, Bern, CH) [10]. It is a software tool for automatic segmentation of brain tumours according to the VASARI guidelines of the National Cancer Institute of the American NIH [11]; we used the results of three tumour region volumes in cubic centimetres (necrotic tissue, active enhancing tumour tissue, and oedema). For comparison with follow-up MRIs, we use the T1contrast image of the first baseline scan of each patient as a reference template [10].

### MRI-based involvement of SVZ and cortex

We used the MRI-based classification of GB based on its participation of the SVZ and cortex [12]: group I, the contrast-enhancing lesion (CEL) extends from the atrium SVZ to the pia; group II, SVZ-contacting a tumour that does not involve the cortex; group III, the CEL is invading the cortex, reaching the pia, but does not touch the SVZ; and group IV, tumours that “respect” both the SVZ and cortex [12].

### ADC measurements

Tumour regions included normal-appearance white matter (NAWM), necrosis, enhancing tumour region, and oedema. Oedema’s ADC was measured two times, at proximal oedema (first 10 mm) and distal oedema (11–20 mm).

We used the BraTumIA segmentations as ROIs for the ADC measurement; then, these ROIs were manually outlined using a pixel-wise application (FuncTool 9.4.04b, GE Healthcare; Magnetic Resonance Unit; Waukesha, Wisconsin, USA): this software generated ADC maps and ADC value measurements of the selected ROIs. We use the mean value of ADC within each ROI as this is the measurement reported by the majority of studies evaluating the prognostic importance of ADC parameters in preoperative imaging [3, 14]; ADC values were expressed in square millimetres per second.

### Definition of progression-free survival

Preoperative MRI acquired before the first operation was the baseline and helped monitor the evolution of the disease. Progression-free survival (PFS) was defined as the time elapsed from radiotherapy completion to disease progression or death. Tumour recurrence was based on the assessment of contrast-enhanced T1-weighted MR images, if it detected a new or progressive increase in enhancing tumour within the initial surgical resection site and a remote location. We also considered RANO criteria to distinguish true progression/recurrence from pseudoprogression when repeat pathology was not available; the clinical diagnosis of pseudoprogression was made if no change in treatment was required for a minimum of 6 months from the end of RT. Progressive disease was defined as a ≥ 25% increase in enhancing illness and clinical deterioration that needed a change in treatment within 6 months after the end of RT [15]. We purposed to evaluate the associations between mean values of ADC parameters and tumour region volumes. Patients were followed until progression was present or until they complete a 30-month period, not until death; then, we did not calculate the overall survival (time elapsed from the completion of radiotherapy to death from any cause).

### Statistical analysis

A one-way analysis of variance (ANOVA) compared the difference of ADC values among tumour regions: normal tissue, enhancing tumour, proximal oedema, distal oedema, and necrosis [16]. We considered two independent variables: tumour regions (within-subjects variable) and the MRI-based involvement of SVZ and cortex (between-subjects variable) and performed a mixed between-within subject ANOVA [17]. This analysis tested whether there were main effects for each of the independent variables and whether the interaction between the two variables was significant. Bonferroni adjustment of the *p* value (.050/5) was set at 0.010; 95% confidence intervals (CI) were calculated according to contemporary definitions [18]. The effect size considered the partial eta squared (*η*^2^) proposed by Cohen [19], *η*^2^ from 0.01 to 0.06 = small effect, *η*^2^ from 0.06 to 0.14 = moderate effect, and *η*^2^ > 0.14 = large effect. Similar analyses were applied to the tumour region volumes.

We performed a partial correlation analysis to calculate the association between ADC values and tumour region volumes. This method estimated correlations without the effect of age and gender; independent reviews were carried out for each tumour region (necrotic tissue, active enhancing tumour tissue, oedema, and NAWM). Correlation coefficients were interpreted as *very strong* (at least of 0.8), *moderately strong* (0.6 up to 0.8), *fair* (0.3 up to 0.6), and *poor* (less than 0.3).

Survival analysis was performed using the Kaplan-Meier method and the log-rank test to compare the PFS curves between subgroups [20]. We calculated Cox’s proportional hazard ratios (HRs) and their adjusted 95% confidence intervals (CIs) [21]. Statistical significance was indicated by a *p* value < 0.05.

Software: all analyses were carried out using the IBM® SPSS® Statistics (software version 23.0.0.2 IBM Corporation; Armonk, NY, USA); box and whisker and correlation plots were produced using Tableau Desktop (software version 8.3.3, Seattle, WA, USA).

## Results

### ADC values across regions

**Fig. 2:**
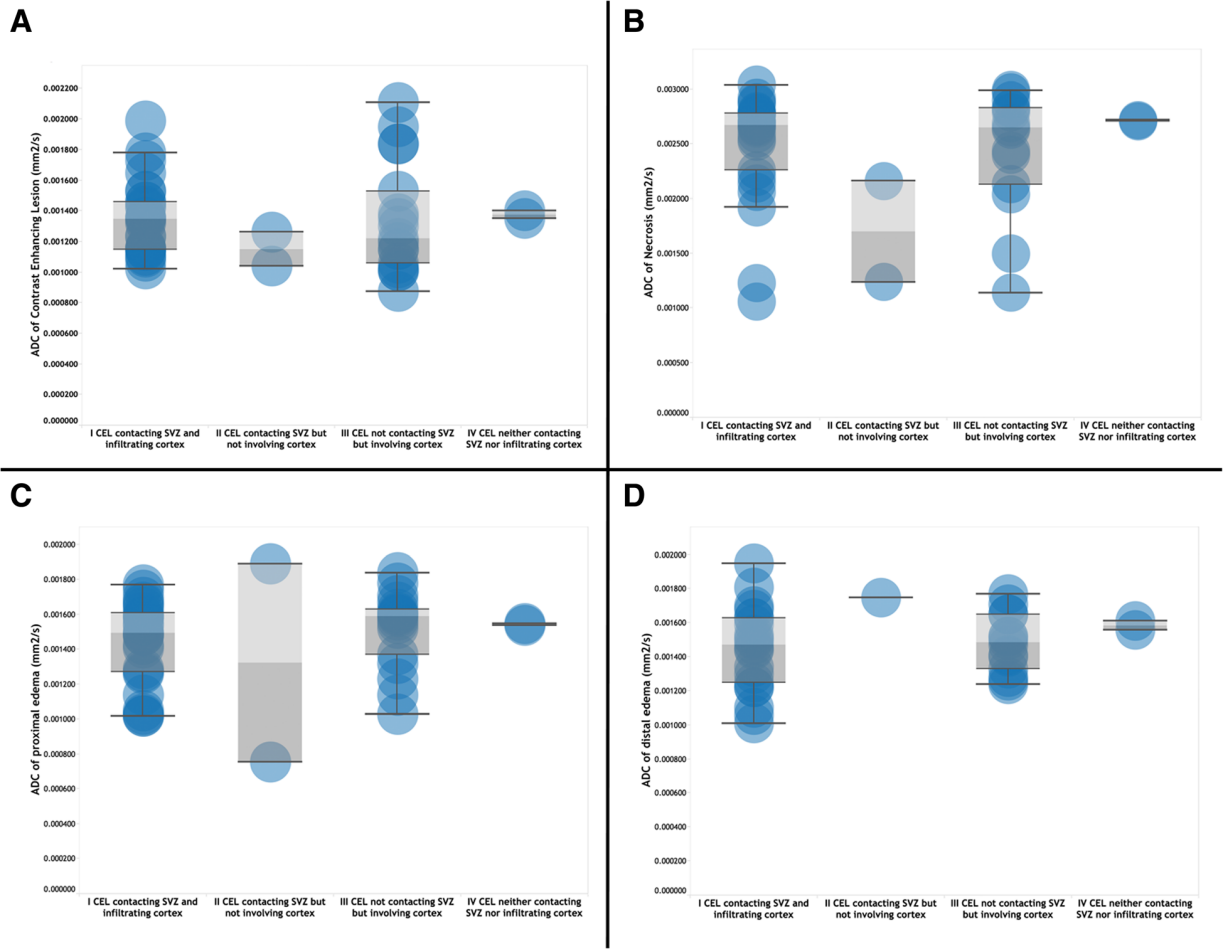
Distribution of ADC values at each tumour region. **a** Contrast-enhancing lesion. **b** Tumour. **c** Proximal oedema. **d** Distal oedema

We found a statistically significant difference in ADC values for the five tumour regions (normal tissue, enhancing tumour, proximal oedema, distal oedema, and necrosis): *F* (4, 220) = 166.71, *p* ≤ .001; the effect size, calculated using eta squared, was .018, meaning a large effect. Post hoc comparisons using the Tukey HSD and Dunnett tests indicated that the mean score for normal tissue was significantly different from the tumour regions and also between ADC values in necrosis compared with the rest of groups (*p* < .001). Table 1 shows means and 95% C.I. of the ADC values for each tumour region; Fig. 2a–d depicts the distribution of ADC values at each tumour region subgrouped by the MRI-based involvement of GB.

**Table 1:** Means and 95% C.I. of the ADC values (mm^2^/s) at each tumour region

	Mean	Std. deviation	Std. error	95% confidence interval	
				Lower bound	Upper bound
Normal tissue ADC	.000770	.000063	.000009	.000752	.000789
Enhancing tumour ADC	.001319	.000344	.000050	.001219	.001418
Proximal oedema ADC	.001468	.000252	.000037	.001394	.001542
Distal oedema ADC	.001474	.000217	.000035	.001403	.001545
Necrosis ADC	.002454	.000523	.000079	.002295	.002613
Total	.001481	.000631	.000042	.001398	.001564

The mixed between-within subjects ANOVA conducted to assess the impact of *MRI-based involvement of SVZ and cortex* on patients’ ADC values across *tumour regions* showed no significant interaction between variables, Wilks’ Lambda = .765, *F* (12, 77) = .683, *p* = .762, and *η*^2^ = .085 (a moderate effect size). There was a substantial main effect for tumour regions, Wilks’ Lambda = .056, *F* (4, 29) = 122.87, *p* < .001, and *η*^2^ = .944 (a large effect size), with groups showing an increment in ADC values across tumour regions. The main effect comparing the MRI-based involvement of SVZ and cortex was not \significant, *F* (3, 32) = .226, *p* = .878, and *η*^2^ = .021 (small effect size), suggesting no influence of the SVZ and cortex involvement by GB [assignment of patients in each group was I, 27 (56.24%); II, 2 (4.17%); III, 17 (35.42%), and IV, 2 (4.17%)]*.* Table 2 shows the estimated marginal means of ADC values grouped by tumour regions and across SVZ and cortex involvement (MRI-based classification).

**Table 2:** Estimated marginal means of ADC values (mm^2^/s) at each tumour regions and subgrouped based on SVZ and cortex involvement (MRI-based classification)

Tumoural zone	Tumour regions	Mean	Std. error	95% confidence interval	
				Lower bound	Upper bound
I CEL contacting SVZ and infiltrating cortex	Normal tissue ADC	.000781	.000061	.000661	.000902
	Enhancing tumour ADC	.001305	.000061	.001185	.001425
	Proximal oedema ADC	.001443	.000062	.001320	.001565
	Distal oedema ADC	.001447	.000069	.001311	.001583
	Necrosis ADC	.002489	.000063	.002364	.002614
II CEL contacting SVZ but not involving cortex	Normal tissue ADC	.000742	.000224	.000301	.001184
	Enhancing tumour ADC	.001150	.000224	.000709	.001591
	Proximal oedema ADC	.001325	.000224	.000884	.001766
	Distal oedema ADC	.001750	.000316	.001126	.002374
	Necrosis ADC	.001700	.000224	.001259	.002141
III CEL not contacting SVZ but involving cortex	Normal tissue ADC	.000753	.000077	.000602	.000905
	Enhancing tumour ADC	.001353	.000077	.001202	.001504
	Proximal oedema ADC	.001515	.000077	.001364	.001667
	Distal oedema ADC	.001479	.000085	.001313	.001646
	Necrosis ADC	.002461	.000082	.002300	.002622
IV CEL neither contacting SVZ nor infiltrating cortex	Normal tissue ADC	.000795	.000224	.000354	.001236
	Enhancing tumour ADC	.001375	.000224	.000934	.001816
	Proximal oedema ADC	.001545	.000224	.001104	.001986
	Distal oedema ADC	.001585	.000224	.001144	.002026
	Necrosis ADC	.002715	.000224	.002274	.003156

### Tumour region volume assessment

Assessment of volumes across tumour regions showed similar results; one-way between-groups ANOVA showed a statistically significant difference for the three evaluated tumour region volumes (necrosis, enhancing tumour, and peritumoural oedema): *F* (2, 141) = 136.3, *p* ≤ .001, and *η*^2^ = .014, meaning a moderate effect. Post hoc comparisons using the Tukey HSD indicated that the only significantly different mean score was peritumoural oedema (*p* < .001), with no significant differences between necrosis and enhancing tumour volumes. Table 3 shows the tumour region volumes (cm^3^) observed in GB.

**Table 3:** Tumour regions volumes (cm^3^) observed in GB

Volumes (cm^3^)	Mean	Std. deviation	Std. error	95% confidence interval	
				Lower bound	Upper bound
Necrosis	8.83	9.11	1.31	6.18	11.47
Enhancing tumour	11.14	10.54	1.52	8.08	14.20
Peritumoural oedema	79.67	38.95	5.62	68.36	90.98
Total	33.21	40.62	3.38	26.52	39.90

A mixed between-within subjects ANOVA conducted to assess the impact of MRI-based involvement of SVZ and cortex on patients’ volumes across three tumour regions showed no significant interaction between variables, Wilks’ Lambda = .811, *F* (6, 86) = 1.584, *p* = .161, and *η*^2^ = .10 (moderate effect size). There was a substantial main effect for tumour regions, Wilks’ Lambda = .505, *F* (2, 43) = 21.055, *p* < .001, and *η*^2^ = .495 (a large effect size), with a gradual increment of volumes between regions. The main effect of MRI-based involvement of SVZ and cortex was not significant, *F* (3, 44) = 1.075, *p* = .370, and *η*^2^ = .068 (small effect size), meaning no influence of the MRI-based classification in tumour volumes*.* Table 4 depicts the tumour region volumes (cm^3^) grouped by tumour regions and across SVZ and cortex involvement (MRI-based classification).

**Table 4:** Estimated marginal means volumes (cm^3^) at each tumour regions and subgrouped based on SVZ and cortex involvement (MRI-based classification)

MRI-based tumour region volumes (cm^3^)	Region	Mean	Std. error	95% confidence interval	
				Lower bound	Upper bound
I CEL contacting SVZ and infiltrating cortex	Necrosis	10.916	1.745	7.399	14.432
	Enhancing lesion	13.526	2.019	9.458	17.595
	Peritumoural oedema	75.106	7.266	60.463	89.749
II CEL contacting SVZ but not involving the cortex	Necrosis	6.056	6.411	− 6.864	18.975
	Enhancing lesion	10.552	7.417	−4.396	25.501
	Peritumoural oedema	31.095	26.696	− 22.707	84.897
III CEL not contacting SVZ but involving the cortex	Necrosis	6.458	2.199	2.027	10.890
	Enhancing lesion	8.109	2.544	2.982	13.236
	Peritumoural oedema	93.442	9.157	74.988	111.896
IV CEL neither contacting SVZ nor infiltrating cortex	Necrosis	3.546	6.411	− 9.374	16.466
	Enhancing lesion	5.352	7.417	− 9.597	20.301
	Peritumoural oedema	72.747	26.696	18.945	126.549

### ADC values and tumour volumes correlate

**Fig. 3:**
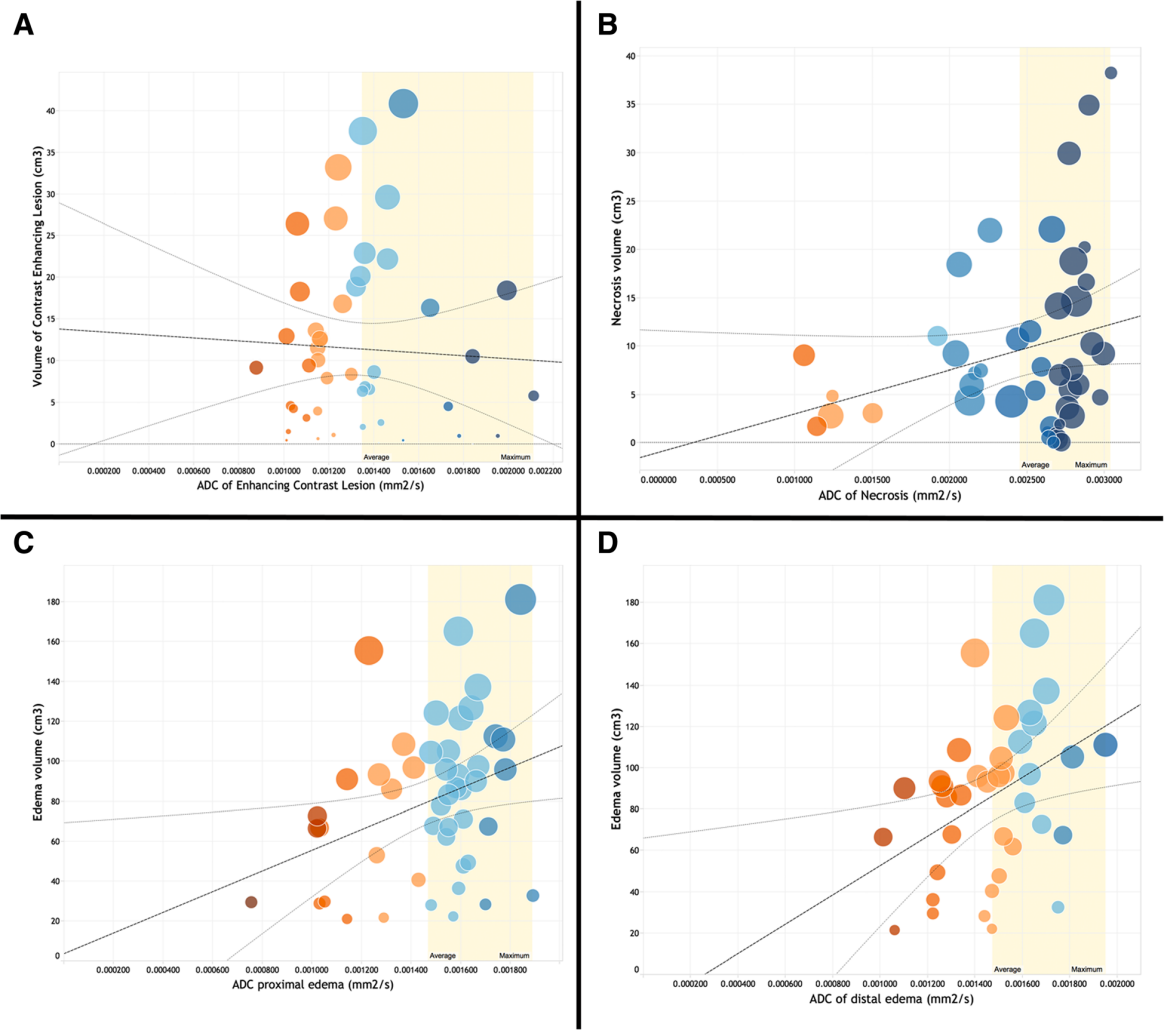
Scatterplots with regression lines of similar association between ADC values and tumour region volumes; a yellow colour band indicates ADC values above the mean. **a** Contrast-enhancing lesion. **b** Tumour. **c** Proximal oedema. **d** Distal oedema

Partial correlation analyses between ADC values and their corresponding tumour region volumes were controlled for age and gender. There was a poor, negative, partial correlation between ADC of the enhancing tumour and its corresponding volume, *r* = −.045, df = 45, and *p* = .767; a fair, positive, partial correlation was depicted between ADC of the proximal oedema and peritumoural volume, *r* = .347, df = 43, and *p* = .019; this fair, positive, association was more significant between ADC of distal oedema and peritumoural volume, *r* = .418, df = 34, and *p* = .011; and a positive, poor, partial correlation was observed between ADC of necrosis and necrosis volume, *r* = .263, df = 4540, and *p* = .093. An inspection of the zero order correlation suggested that controlling for age and gender increased the significance of correlations between ADC values in oedema and peritumoural volumes. Figure 3 depicts scatter plots between ADC values and peritumoural regions.

### Progression-free survival analysis

**Fig. 4:**
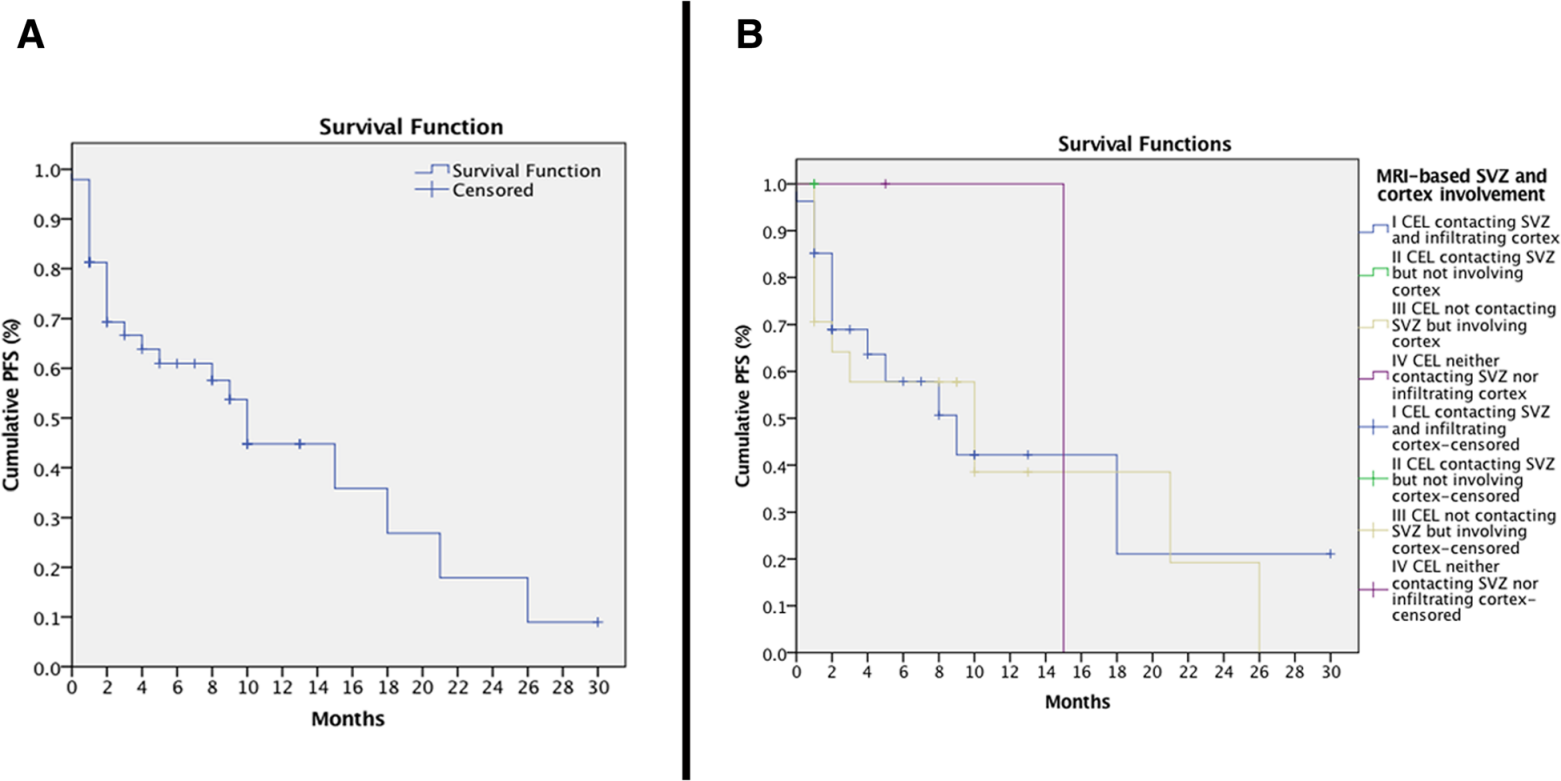
Curves for progression-free survival. **a** Progression-free survival curve of the whole sample. **b** Progression-free survival curves grouped by MRI-based subventricular zone and cortex involvement by GB

The median PFS time was 10 months (SE 1.402, C.I. 7.253–12.747, Fig. 4a); after grouping by SVZ and cortex involvement, there were no statistical differences between the PFS curves, *χ*^2^ (3) = .673 and *p* = .879; Fig. 4b. A Cox regression omnibus test of model coefficients was not significant for the tumour region ADC values: − 2 log-likelihood = 113.010, *χ*^2^ (4) = 4.166, and *p* = .384. A second Cox proportional hazards regression analysis considering only tumour region volumes provided an almost significant model: − 2 log-likelihood = 146.066, *χ*^2^ (4) = 9.303, and *p* = .054. Only the non-enhancing tumour volume depicted a trend towards significance of the hazard function: *p* = .067 and HR = 1.077. This value indicated that, for every additional unit increase in cubic centimetres of a non-enhancing tumour, patients increased 7.7% of the risk to report a tumour progression, controlling for all other factors in the model.

## Discussion

Although the longstanding interest for gliomas is the identification of prognostic markers [22], the mean GB survival reaches only 14 months [23]. There has been a growing interest in the study of tumour region volumes extracted from the routine MR examination with an emphasis in oedema boundaries [24, 25], as well as the use of several diffusion-derived biomarkers in the differentiation [26] and tumour progression in GB [27].

The relevance of this study has several components: first, we observed a significant difference among ADC values on MRI-based locations of GB (ADC values higher in distal oedema). Second, we manifest that there is not a substantial effect of the MRI-based involvement of SVZ and cortex by GB in ADC measurements. Third, our multivariate assessment of volumes showed a statistically significant difference among tumour regions (necrosis, enhancing tumour, and peritumoural oedema), with significant post hoc difference only for peritumoural oedema. We also found a substantial correlation between proximal and distal ADC values with the peritumoural volumes; these facts agree with recent studies that accept pre-treatment diffusion MRI as a predictive imaging biomarker (specific diffusion signature) associated with particular survival [28] and single nucleotide polymorphism [29].

Despite that there are dozens of recent studies showing the usefulness of several ADC-derived parameters for tumour differentiation and survival in GB [30, 31], an analysis considering ADC values at different tumour regions, and their associated volumes, together with PFS, is scarce in the literature, to the best of our knowledge. Besides, there is a lack of consistent threshold values to differentiate tumour progression from necrosis [32], and our reported ADC values grouped by tumour regions contribute to filling this gap. The use of segmented volumes is relevant, as even the improved RANO criteria fall short of definitively distinguishing tumour progression (it uses only the sum of the products of perpendicular diameters of enhancing lesions) [15]. Recent studies report survival analysis in GB using measurements of diameters in peritumoural oedema [33]; this method, in our perspective, has a strong potential to underestimate the actual size of peritumour regions.

Report of ADC values is not standard, but can have a relevant meaning in the follow-up of treatment response in GB; recent studies have proved a significant association of ADC in subgroups stratification [34], the methylation status of the MGMT promoter [35], and its predictive value in overall survival [34]. The study by Romano et al. [35] may look similar to our research. However, they did not perform a partial correlational analysis comparing volumes and ADC regions, neither did they consider separate study based on tumour regions. Peritumoural oedema in MR plays a role as an independent prognostic factor. Schoenegger et al. [25] found that patients with the presence of severe oedema had shorter overall survival significantly compared to patients with minor oedema. Also, distant oedema has been correlated with a higher degree of necrosis and vascular endothelial growth factor (VEGF) expression [24].

Gliomas produce microscopic invasion to surrounding tissues, especially white matter (WM) tracts [36], from the visible area of disease [37]; as a result, there is NAWM, where infiltration is not able to be detected using conventional MRI protocols [38]. Peritumoural oedema on T_2_-weighted imaging (T_2_-w) is often assumed to signal penetration into the brain tissue [39]. We found that the non-enhancing tumour volume depicted a trend towards significance of the hazard function of 7.7%, meaning that for every additional unit increase in cubic centimetres of the non-enhancing tumour region, patients increased 7.7% of the risk to report a tumour progression. Pre-treatment peritumoural oedema is also associated with an increased risk of the development of incremental oedema after stereotactic radiosurgery (SRS), in many cases causing a worsening of the clinical status [40].

Some limitations of this study need to be mentioned: the data were analysed retrospectively, so a control group was not available. Several clinical and surgical variables were intentionally out of the scope of this study (extent of resection, radiation dose, and adjuvant chemotherapy). As part of a research line investigation, the potential of quantitative MR biomarkers in brain tumours, this study focused on selected variables, and we acknowledge this study deserves a sequel of additional findings. Also, for this group of patients, we did not have access to advanced biomarkers such as spectroscopy, perfusion, and diffusion tensor imaging techniques. We would have liked to use MRI perfusion for the differentiation of true progression/recurrence from pseudoprogression, considering that many other studies proposed this technique as an affordable and valuable diagnostic tool. Future prospective studies could regard perfusion as an additional method to be performed. However, none of these advanced MRI biomarkers is used to anticipate tumour progression on a day-to-day basis [41, 42]. Instead, we used the standard T1 and T2 imaging features which remain as the criterion standard [43], as well as commercially available software for ADC measurements which may facilitate reproducibility of our findings.

In conclusion, specific ADC values at selected tumour regions (distal oedema) is a quantifiable predictive biomarker in the PFS of patients with GB. This parameter might offer clinical applicability to clinicians in the short term and routine assessment in GB.

## Abbreviations

ADC: Apparent diffusion coefficient
ANOVA: Analysis of variance
CEL: Contrast-enhancing lesion
CI: Confidence intervals
DWI: Diffusion-weighted imaging
GB: Glioblastoma
MGMT: O^6^-methylguanine-DNA methyltransferase
MR: Magnetic resonance
MRI: Magnetic resonance imaging
NAWM: Normal-appearance white matter
PFS: Progression-free survival
SRS: Stereotactic radiosurgery
SVZ: Subventricular zone
VEGF: Vascular endothelial growth factor
WM: White matter
*η*^2^: Partial eta squared
